# Linking metabolism and metastasis: elevated α-hydroxybutyric acid in oral squamous cell carcinoma patients with lymph node metastasis

**DOI:** 10.1007/s11306-026-02431-7

**Published:** 2026-04-18

**Authors:** Xiaolian Gu, Philip J. Coates, Lixiao Wang, Nicola Sgaramella, Mustafa Magan, Karin Nylander

**Affiliations:** 1 Department of Medical Biosciences/Pathology, Umeå University, Building 6M, 2nd floor, Analysvägen 9, Umeå, 901 87 Västerbotten Sweden; 2 Research Centre for Applied Molecular Oncology (RECAMO), Masaryk Memorial Cancer Institute, Brno, 656 53 Czech Republic; 3 Department of Oral and Maxillo-Facial Surgery, Mater Dei Hospital, Bari, 701 25 Italy; 4 Department of Clinical Sciences/ENT, Umeå University, Umeå, 901 87 Västerbotten Sweden

**Keywords:** Oral cancer, Plasma, Metabolomics, Glucose, α-hydroxybutyric acid

## Abstract

**Introduction:**

Metabolic reprogramming is a hallmark of cancer. Plasma metabolomics offers a minimally invasive approach for identifying metabolic alterations that may provide insights into tumor progression.

**Objectives:**

We aimed to characterize plasma metabolomic profiles in patients with oral squamous cell carcinoma (OSCC) and evaluate their clinical relevance.

**Methods:**

Plasma samples from 43 OSCC patients and 129 cancer-free controls, matched at a 1:3 ratio based on age, sex, and body mass index, were analyzed using gas chromatography-mass spectrometry (GC-MS). A random forest algorithm was applied to identify key metabolic features distinguishing OSCC from controls. The clinical significance of the top metabolites was assessed and validated in another OSCC cohort (*n* = 27).

**Results:**

A total of 113 compounds were putatively annotated and analyzed based on relative abundances. A ten-feature panel demonstrated good classification performance (area under the curve = 0.87; Matthews correlation coefficient = 0.703). The ten features are maltose, glucose, xylulose, δ-gluconolactone, fructose, indoleacetic acid, α-hydroxybutyric acid, glutamic acid, cysteine, and the monoacylglyceride MG(18:1(9Z)/0:0/0:0), suggesting dysregulated carbohydrate metabolism and oxidative stress as the major plasma metabolomic alterations in OSCC. Notably, α-hydroxybutyric acid levels were elevated in patients with regional lymph node metastasis compared with those without.

**Conclusion:**

Our findings underscore the intricate interplay between altered glucose metabolism, redox imbalance, and OSCC. α-hydroxybutyric acid, a marker of oxidative stress and an indicator of insulin resistance, may be associated with metastatic progression.

**Supplementary Information:**

The online version contains supplementary material available at 10.1007/s11306-026-02431-7.

## Background

Metabolomics, the high-throughput study of small-molecule metabolites in biological systems, has emerged as a powerful tool for uncovering the biochemical alterations associated with disease (Danzi et al., 2023; Wishart, 2016). Since metabolites represent the intermediates and end-products of cellular processes, metabolomic profiling provides a snapshot of an organism’s physiological state, reflecting both intrinsic and extrinsic influences such as genetic/epigenetic alterations, nutrients, drugs, lifestyle, and environmental conditions (Gupta et al., 2024; Schmidt et al., 2021). Metabolic reprogramming is a hallmark feature of cancer, with tumors often exhibiting alterations in pathways related to energy production, lipid metabolism, and amino acid biosynthesis (Gupta et al., 2024; Schmidt et al., 2021). These metabolic changes can be detected in biofluids such as plasma, making metabolomics a promising approach for non-invasive cancer biomarker discovery (Chen et al., 2024; Irajizad et al., 2023; Wang et al., 2023).

Oral squamous cell carcinoma (OSCC) is the most common type of oral cancer, arising from squamous epithelial cells lining the oral cavity (Tan et al., 2023). While metabolomic studies have provided insights into the biochemical alterations in various cancers, the metabolic profile of patients with OSCC remains less well-characterized. Saliva, serum, plasma, urine, and tissue samples from OSCC patients have been investigated, with findings showing considerable variability regarding the specific metabolites and metabolic pathways implicated in oral carcinogenesis, due to differences in study design, analytical platforms, and/or data processing methods (Alapati et al., 2023; Luo et al., 2024; Vitorio et al., 2020). This lack of consistency underscores the need for further research to identify robust and reproducible metabolic biomarkers for OSCC.

Among the various analytical platforms available, gas chromatography-mass spectrometry (GC-MS)-based metabolomic technologies are widely employed due to their high sensitivity, resolution, and ability to detect a wide range of volatile and semi-volatile metabolites. With appropriate derivatization, they can also detect metabolites that are inherently non-volatile, thereby enabling the reliable analysis of a broad spectrum of metabolites involved in central metabolic pathways (Danzi et al., 2023). In this study, targeted GC-MS was employed to profile plasma metabolites in patients with OSCC, aiming to uncover underlying metabolic alterations that could support the development of novel biomarkers for early detection and disease monitoring.

## Materials and methods

### OSCC patients (1st patient cohort) and matched controls

Forty-three patients diagnosed with OSCC at Norrland’s University Hospital in Umeå, Sweden were recruited. Blood samples were collected in heparin tubes prior to treatment. Plasma was aliquoted and stored at -80 °C within 1 h of collection. A total of 129 cancer-free individuals were matched to the 43 patients at a 3:1 ratio based on age, sex, and body mass index (BMI). Heparin plasma samples from the cancer-free individuals were obtained from Biobank Norr, Umeå. For these samples, blood collection and processing were performed following a standardized protocol. Briefly, after a minimum of 8 h of fasting, blood samples were collected in heparin tubes in the morning. Plasma samples were aliquoted and frozen within 1 h of collection, either directly at -80 °C or at -20 °C for up to one week before being transferred to a central storage facility.

### OSCC patients without matched controls (2nd patient cohort)

An additional cohort of 27 OSCC patients was recruited. No cancer-free controls were matched to these patients. Blood samples were collected in heparin tubes prior to treatment. Demographic and clinical features of both patient cohorts are presented in Table 1. Except for differences in age and fasting status, the characteristics of patients in the two cohorts were comparable.

**Table 1:** Demographic and clinical features of controls and the two patient cohorts

		Control	Patient		*p*-value (Fisher’s exact test)	
			1st cohort (control-matched)	2nd cohort (non-matched)	Control vs. 1st cohort	1st vs. 2nd cohort
Age	≤ 40 years	39	13	0	1	< 0.001
	> 40 years	86	29	27		
Sex	Female	44	15	14	1	0.218
	Male	81	27	13		
Obesity	No	88	30	22	1	0.403
	Yes	37	12	5		
Hypertension	No	71	31	22	0.067	0.565
	Yes	54	11	5		
Diabetes^*^	No	108	36	25	0.070	0.467
	Yes	5	6	2		
Fasting status	Fasting	125	31	26	<0.001	0.021
	Non-fasting	0	11	1		
Tumor subsite	Oral tongue		29	13		0.129
	Other oral subsites		13	14		
Tumor size	T1, T2		28	13		0.141
	T3, T4		14	14		
lymph node metastasis	Negative		27	18		1.000
	Positive		15	9		
TNM stage	I, II		21	11		0.471
	III, IV		21	16		

*OSCC* oral squamous cell carcinoma

*Diabetes status was unavailable for 12 controls

### Ethics

This study was approved by the Regional Ethics Review Board, Umeå, Sweden (Dnr 08-003 M, 2012-131-33 M and 2024-00725-02) and conducted in accordance with the Declaration of Helsinki. Written informed consent was obtained from all participants.

### Metabolomics analysis

Plasma samples were sent to The Swedish Metabolomics Centre in Umeå, Sweden (www.swedishmetabolomicscentre.se) for metabolic profiling using Gas Chromatography - Time-of-Flight - Mass Spectrometry (GC-TOF-MS). Each set of case and matched control samples was measured consecutively to minimize measurement order bias. Non-matched case samples were measured after all case-control sets. Metabolite annotation and quantification were performed jointly for samples from both cohorts.

Internal standards alpha-ketoglutarate-13C4, myristic acid-13C3, cholesterol-D7 were obtained from Cil (Andover, MA, USA). Succinic acid-D4, hexadecanoic acid-13C4, D-glucose-13C6, D-sucrose-13C12 were obtained from Sigma (St. Louis, MO, USA). Metabolites were extracted by mixing 80 µL plasma with 720 µL 90:10 (v/v) methanol: water solution containing internal standards. Each plate was extracted for 30 s using a mixing script on an Agilent Bravo automated liquid handling platform, incubated in the freezer at -20 °C for 2 h and centrifuged at 4 °C 14,000 RPM, for 10 min. Derivatization and GC-MS analysis were performed as described previously (Gullberg et al., 2004) with the samples being derivatized in 15 µl methoxyamine (15 µg/µl in pyridine) and 30 µl of a 1:1 mix of MSTFA (+ 1% TMCS) and heptane including methyl stearate (15 ng/µl). 1 µL of the derivatized sample was injected in splitless mode by a L-PAL3 autosampler (CTC Analytics AG, Switzerland) into an Agilent 7890B gas chromatograph equipped with a 10 m x 0.18 mm fused silica capillary column with a chemically bonded 0.18 μm Rxi-5 Sil MS stationary phase (Restek Corporation, U.S.) The injector temperature was 270 °C, the purge flow rate was 20 mL min-1 and the purge was turned on after 60 s. The gas flow rate through the column was 1 mL min-1, the column temperature was held at 70 °C for 2 min, then increased by 40 °C min-1 to 320 °C, and held there for 2 min. The column effluent was introduced into the ion source of a Pegasus BT time-of-flight mass spectrometer, GC-TOF-MS (Leco Corp., St Joseph, MI, USA). The transfer line and the ion source temperatures were 250 °C and 200 °C, respectively. Ions were generated by a 70 eV electron beam at an ionization current of 2.0 mA, and 30 spectra s-1 were recorded in the mass range m/z 50–800. The acceleration voltage was turned on after a solvent delay of 150 s. The detector voltage was 1800–2300 V.

All non-processed MS-files were exported from the ChromaTOF software in NetCDF format to MATLAB R2023a (Mathworks, Natick, MA, USA), where all data pre-treatment procedures, such as base-line correction, chromatogram alignment, data compression and Multivariate Curve Resolution were performed. The extracted mass spectra were identified by comparisons of their retention index and mass spectra with libraries of retention time indices and mass spectra (Schauer et al., 2005) using NIST MS 2.2 software. Compound annotation was based on forward and reverse spectral matching against a curated in-house GC-MS metabolite library developed at the Swedish Metabolomics Centre, Umeå University. Masses and ratio between masses indicative of a derivatized metabolite were especially notified. The mass spectrum with the highest probability indicative of a metabolite and the retention index between the sample and library for the suggested metabolite was ± 5 (usually less than 3) the deconvoluted “peak” was annotated as an identification of a metabolite. Metabolite annotations therefore correspond to Metabolomics Standards Initiative (MSI) level 2 (putatively annotated compounds) and were manually reviewed by experienced analysts at the Swedish Metabolomics Centre. Chromatographic peaks were resolved using spectral deconvolution, and for each metabolite a compound-specific fragment ion was selected for quantification. Metabolite abundances were calculated as the integrated peak area of the selected fragment ion. Internal standards (IS02 and IS07; Table S1) were added prior to extraction and derivatization to monitor analytical performance. Seven pooled quality control (QC) samples were included in the analytical batch to assess analytical stability and reproducibility during the measurement sequence. As the analysis provides relative quantification, metabolite abundances were scaled so that the maximum value for each metabolite across samples was set to 100 to facilitate comparison of relative changes among samples.

### Data analysis

Metabolomic data were analyzed using MetaboAnalyst, a web-based platform for comprehensive metabolomics data analysis (Ewald et al., 2024; Pang et al., 2024). Original relative abundance data from the first cohort were uploaded and normalized using log transformation (base 10) and auto-scaling. To identify differences between patients and matched controls, univariate analysis was performed using t-tests, with *p*-values adjusted for multiple comparisons using the Benjamini–Hochberg false discovery rate (FDR) method. Principal component analysis (PCA) was used for linear dimensionality reduction, and the Random Forest algorithm was applied to capture potential non-linear relationships (Greener et al., 2022). All analyses were performed using the normalized data and default parameters in MetaboAnalyst. Model performance was evaluated using cross-validation, as implemented in MetaboAnalyst. In addition, model stability was assessed using the out-of-bag (OOB) error estimate inherent to the Random Forest algorithm, which provides internal validation based on bootstrap resampling.

For analyses relating clinical features to metabolomic alterations, original relative abundance data were used. Associations between two binary variables were assessed using Fisher’s exact test. To compare numerical variables between two groups of samples, the non-parametric Mann-Whitney U test was used. Survival analysis was performed using the Kaplan‑Meier method and Cox-regression. Two survival measures were assessed: overall survival (time from diagnosis to death from any cause) and disease‑free survival (time from completion of treatment to relapse or death). Statistical tests were conducted in IBM SPSS Statistics 28 (IBM Corp., Armonk, NY, USA). A two-sided *p*-value < 0.05 was considered statistically significant.

Box plots were created using ggplot2 (R version 4.5.0).

## Results

### GC-TOF-MS data

A total of 199 samples were processed for metabolomics profiling. However, samples from one patient in the first cohort and one control could not be analyzed. To maintain the intended case–control matching, the three control samples matched to the failed patient sample were excluded. As a result, 194 samples were included in the final data analysis: 42 patients from the first cohort, 125 matched controls, and 27 patients from the second cohort (Table S1). PCA analysis of all samples and seven QC samples indicated no major analytical issues, although the first five QC samples clustered closely while the last two were more distant, reflecting a measurement order bias (Fig. S1). The non-matched cases from the second patient cohort were measured after all case-control sets from the first cohort; therefore, the two patient cohorts were not compared directly. In total, 113 compounds were annotated, including organic acids, lipids, and organic oxygen compounds (Fig. S2).

### Drug-related metabolites

Statistically significant differences in 38 compounds were identified between 42 patients and the 125 matched controls using t-test (adjusted *p*-value < 0.05, Table 2). Notably, patients exhibited higher plasma levels of acetaminophen compared to controls. Acetaminophen is a synthetic drug commonly used for pain relief and fever reduction, and we identified that a further seven compounds were highly correlated with acetaminophen (*r* > 0.5). These compounds correspond to the top eight differentially abundant metabolites between patients and controls. Among these, *p*-cresol glucuronide is a conjugated metabolite of *p*-cresol, sharing primary metabolic pathways with acetaminophen. Capric acid is used in various nutritional supplements and medical formulations, and tricaprylin, glyceryl 2-caprate dicaprylate, and glyceryl 2-caprylate dicaprate are lipid-based excipients commonly employed in drug formulations to enhance solubility and bioavailability of poorly water-soluble drugs.

**Table 2:** Differentially abundant compounds between patients and controls

Compound	Fold change (patient/control)	Adjusted *p*-value
p-Cresol glucuronide	2872.40	5.36E-22
Acetaminophen	12.14	5.36E-22
Isomaltose	71.46	3.93E-17
Capric acid	7.84	1.37E-13
Tricaprylin	154.04	3.17E-12
Glyceryl 2-caprylate dicaprate	105.94	3.19E-12
Glyceryl 2-caprate dicaprylate	200.13	3.56E-12
δ-tocopherol	3.98	5.66E-08
Maltose	1.95	7.25E-08
Xylulose	1.37	3.50E-07
Glucose	1.13	3.97E-07
Fructose	2.62	6.73E-06
α-hydroxybutyric acid (2-HBA)	1.57	6.90E-06
Indoleacetic acid (IAA)	0.68	1.03E-05
δ-gluconolactone	2.07	2.69E-05
MG(18:1(9Z)/0:0/0:0)	1.71	2.69E-05
Glutamic acid	1.55	9.89E-05
Sorbitol	8.77	5.27E-04
Cysteine	0.87	6.70E-04
Maltotriose	1.62	7.50E-04
Xylitol	3.31	8.19E-04
Threonic acid	1.29	8.19E-04
α-Tocopherol	0.82	8.91E-04
Glycerol 3-phosphate	1.18	1.26E-03
Hypoxanthine	1.43	1.82E-03
Erythritol	1.31	1.93E-03
Lactic acid	1.16	1.93E-03
α-hydroxyisovaleric acid	1.47	2.28E-03
MG(16:0/0:0/0:0)	1.06	5.50E-03
Hippuric acid	0.62	5.59E-03
Arginine	0.79	8.36E-03
Oxoglutaric acid	1.23	1.14E-02
1,5-anhydrosorbitol (1,5-AG)	0.89	1.51E-02
Niacinamide	1.38	1.90E-02
Glucaric acid	16.23	1.94E-02
β-hydroxybutyric acid (3-HBA)	1.82	2.70E-02
Citric acid	0.89	2.84E-02
Benzoic acid	1.09	4.98E-02

Traces of ibuprofen, widely used for pain and fever, and metformin, prescribed for type 2 and gestational diabetes, were detected in certain individuals. However, no significant differences in their levels were observed between patients and controls.

Since the use of Simvastatin (*n* = 4), Levaxin (Levothyroxine, *n* = 6), and Metformin (*n* = 4) was known in a few patients, we further investigated the correlation between intake of these drugs and plasma levels of significantly altered metabolites. No significant associations were identified (data not shown).

To identify endogenous cancer-associated metabolomic changes, all drug or drug-related compounds were excluded from subsequent analysis. Based on the remaining 103 metabolites, a volcano plot illustrating the overall differences in metabolite abundance between patients and matched controls is shown in Fig. S3.

### Principal component analysis (PCA)

PCA based on the 103 metabolomic features revealed similar profiles between the 42 patients and the matched 125 controls (Fig. S4).

### Random forest modeling

**Fig. 1:**
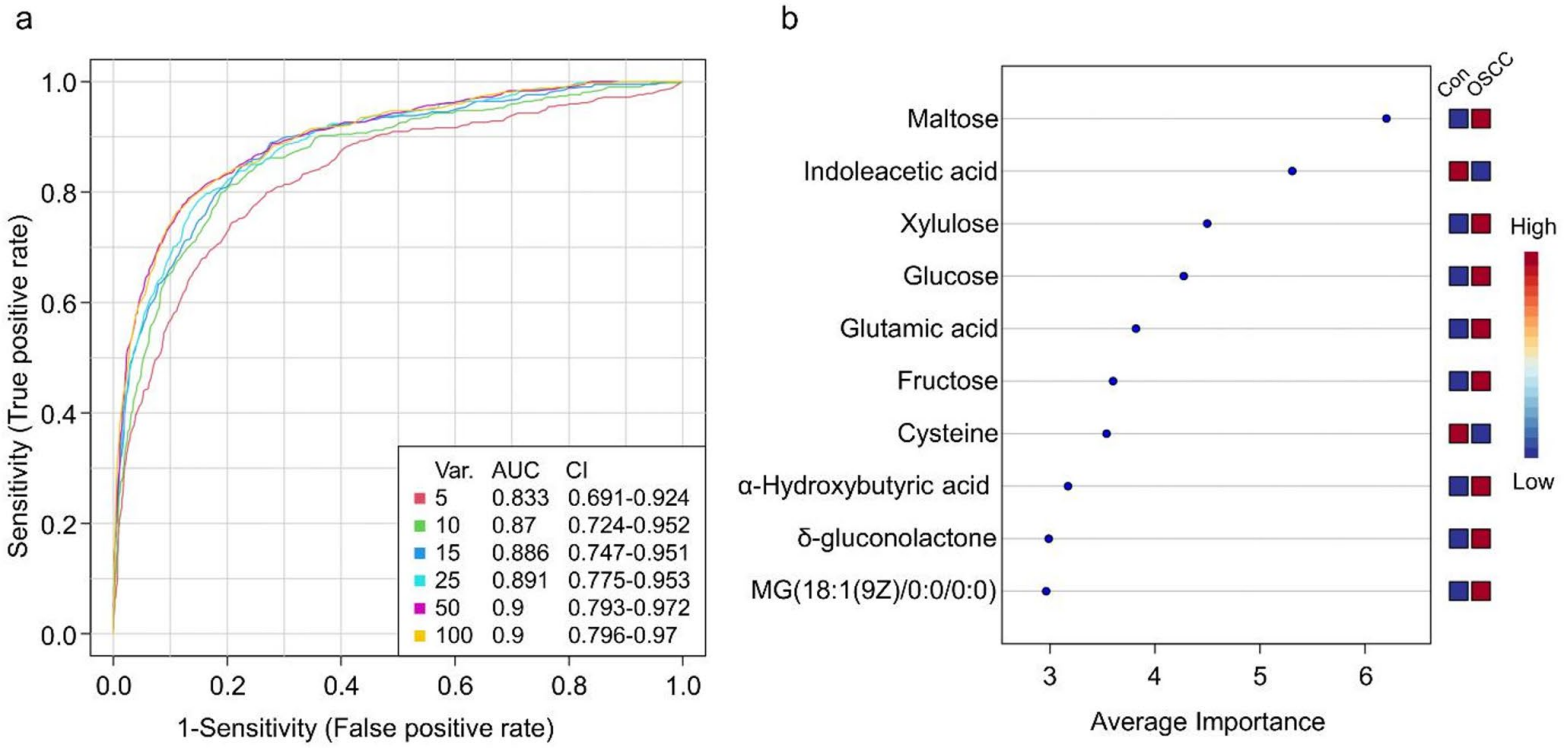
Random forest modeling. **a** ROC curves and their AUC values based on cross-validation performance showing the performance of six models using 5 to 100 metabolomic features. **b** Top 10 significant features ranked by average importance measure. *ROC* receiver-operating characteristic; *AUC* area under the curve. *OSCC* oral squamous cell carcinoma. *Con* matched cancer-free controls

We explored metabolomic changes in the 42 patients using the random forest classification method. Metabolites were ranked according to their importance scores, and the top N metabolites (*N* = 5, 10, 15, 25, 50, or 100) were sequentially selected to evaluate model performance across different feature set sizes. For each feature set, ROC (receiver operating characteristic) curves were plotted to illustrate model performance. The AUC (area under the curve) values of the six models ranged from 0.833 to 0.900 (Fig. 1a). We further evaluated the performance of the six models using the Matthews correlation coefficient (MCC), a metric particularly well-suited for imbalanced datasets. MCC considers all four outcomes of the confusion matrix: true positives, false positives, true negatives, and false negatives (Chicco & Jurman, 2020). Although the 50-feature model showed slightly higher performance for some metrics, the 10-feature model achieved the highest MCC value of 0.703 (Table S2), indicating the most balanced classification performance between the two classes. In addition, the smaller feature set improves model interpretability and reduces the risk of overfitting. Therefore, the 10-feature model was selected as the final model.

KEGG (Kyoto Encyclopedia of Genes and Genomes) pathway analysis based on the top 10 important features (Fig. 1b) identified “starch and sucrose metabolism” as the only significantly enriched pathway (FDR = 0.008). Three metabolites (maltose, glucose, and fructose) were involved in this pathway (Fig. S5). Sub-class chemical structure enrichment analysis revealed significant enrichment of “Carbohydrates and carbohydrate conjugates”, which included maltose, glucose, xylulose, δ-gluconolactone and fructose. All five metabolites exhibited increased plasma levels in patients compared to controls.

**Fig. 2:**
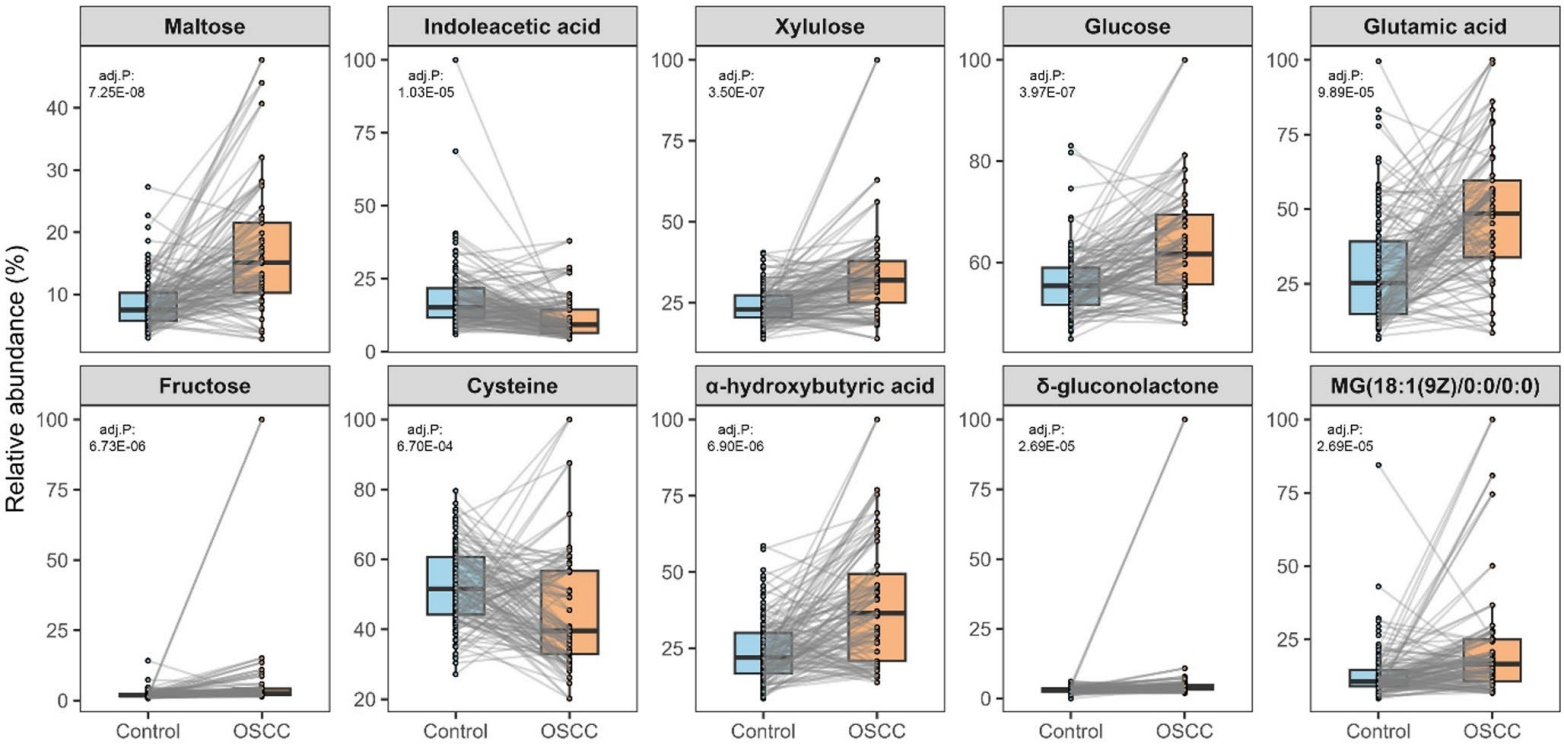
Paired sample box plots showing plasma levels of the top 10 important metabolic features in patients with oral squamous cell carcinoma (OSCC) and matched controls. Adj. P: adjusted p-value

Other metabolites among the top ten included two organic acids (indoleacetic acid and alpha-hydroxybutyric acid), two amino acids (glutamic acid and cysteine) and one lipid molecule (MG(18:1(9Z)/0:0/0:0)). α-hydroxybutyric acid, glutamic acid and MG(18:1(9Z)/0:0/0:0) showed increased levels, whereas indoleacetic acid and cysteine showed decreased levels in patients compared to controls. The paired sample box plots illustrate consistent patterns of changes in most case-control pairs (Fig. 2).

### Clinical features and metabolomic alterations

**Fig. 3:**
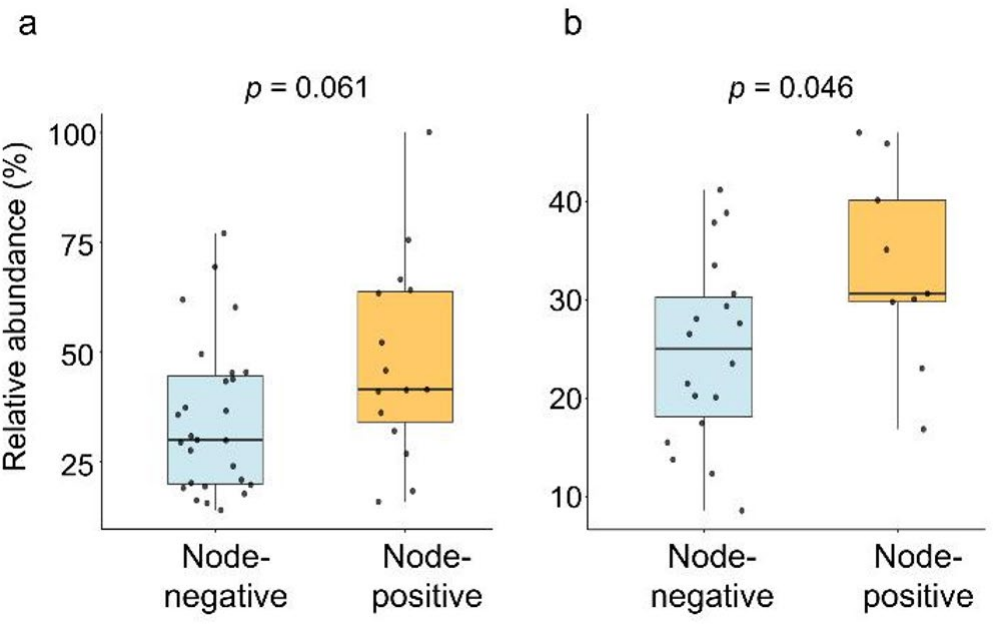
Box plots showing plasma levels of α-hydroxybutyric acid in node-negative and node-positive patients. **a** 1st patient cohort. **b** 2nd patient cohort. Node-negative: patients without regional lymph node metastasis; Node-positive: patients with regional lymph node metastasis

The top ten metabolites were analyzed for differences in abundance related to age (young vs. old), sex (female vs. male), fasting status (fasted vs. non-fasted), tumor subsite (oral tongue vs. other oral subsites), tumor size (T1/T2 vs. T3/T4), and nodal status (negative vs. positive) in the first patient cohort, with findings validated in the second cohort. The two patient cohorts were analyzed separately to account for potential batch effects related to measurement order. Given the limited sample sizes, we adopted a conservative approach, focusing on metabolites that exhibited consistent trends across cohorts. Overall, Mann-Whitney U tests revealed no significant differences in metabolite levels based on age, sex, fasting status, tumor subsite or tumor size (Table S3). However, for α-hydroxybutyric acid, levels tended to be higher in patients with lymph node metastasis compared with those without in the first cohort (*p* = 0.061, effect size = 0.35, 95% confidence interval = 0.000025–0.628) and confirmed as significantly elevated in the second cohort (*p* = 0.046, effect size = 0.48, 95% confidence interval = 0.054–0.76, Fig. 3). None of the metabolites demonstrated a significant impact on overall or disease-free survival using Kaplan-Meier or Cox regression survival analysis.

## Discussion

Blood samples were collected at the time of diagnosis, before any cancer treatment had begun. However, we found that levels of drugs and drug-related compounds were significantly higher in patients compared to controls, such as acetaminophen and p-Cresol glucuronide. These increases did not correlate with the reported use of Simvastatin, Levaxin (Levothyroxine) or Metformin by a few patients, which are typically used to lower cholesterol, treat hypothyroidism and control glucose levels, respectively. This suggests that some patients may have engaged in unreported self-medication, possibly to manage pain or improve their health condition. It has previously been shown that drug metabolites detected in plasma frequently do not align with participants’ self-reported medication use (Suhre et al., 2022). Therefore, unreported self-medication could represent a confounding factor in metabolomics studies. To focus on endogenous cancer-associated metabolomic changes, drug and drug-related compounds were thus excluded from further analysis.

After removing drug-related compounds, our ten-feature random forest model of endogenous metabolites showed good performance in discriminating 42 OSCC from 125 matched controls. Five of the ten features were “carbohydrates and carbohydrate conjugates”, including maltose, glucose, xylulose, δ-gluconolactone and fructose. A common phenomenon in cancer is the Warburg effect, where tumor cells increase their rate of glucose uptake and rely on glycolysis for energy production even in the presence of sufficient oxygen. This metabolic shift results in lower extracellular glucose levels and higher lactate concentrations within the tumor microenvironment (Fendt, 2024; Finley, 2023; Hardie, 2022). Studies analyzing tissue, serum, or saliva samples have reported significantly decreased glucose levels in OSCC patients compared to healthy controls (Enomoto et al., 2018; Ogawa et al., 2014; Song et al., 2020; Tsai et al., 2020). However, in accordance with our findings, an increase in serum glucose levels in patients compared to controls has also been reported (Tiziani et al., 2009; Yang et al., 2022), and elevated plasma glucose was seen in association with cancer development in a rat oral cancer model (Kong et al., 2015). These observations align with evidence suggesting a complex interplay between cancer progression and systemic metabolic alterations (Swanton et al., 2024). While tumor cells exhibit increased glucose uptake locally, the altered metabolic environment can influence overall glucose homeostasis. One possible explanation for the increased plasma glucose levels is the presence of cancer-associated insulin resistance (Dev et al., 2018; Marmol et al., 2023; Masi & Patel, 2021). This phenomenon may be further compounded by stress-induced hyperglycemia (Dungan et al., 2009; Kanter et al., 2024).

Maltose is a disaccharide composed of two glucose molecules. Xylulose and δ-gluconolactone are intermediates in the Pentose Phosphate Pathway (PPP), which supports nucleotide synthesis and redox balance in cancer cells (Patra & Hay, 2014). Fructose is a monosaccharide that is often formed by cleavage of sucrose into fructose and glucose. Overall, increased plasma levels of several common dietary sugars (glucose, fructose, and maltose) and sugar derivatives involved in specific metabolic reactions (xylulose and δ-gluconolactone) indicate that alterations in the metabolism of simple carbohydrates represent the major systemic change in OSCC metabolites.

Alterations in three metabolites related to glutathione synthesis, a critical pathway for cellular antioxidant defense and redox homeostasis (Lapenna, 2023), were also uncovered in patients: α-hydroxybutyric acid, glutamic acid, and cysteine. α-hydroxybutyric acid and glutamic acid levels were elevated while cysteine levels were decreased in patients. Glutamic acid, known as the primary nutrient source to support tumor growth, is among the most frequently dysregulated metabolites in OSCC patients (Alapati et al., 2023). α-hydroxybutyric acid, a byproduct of cysteine synthesis during glutathione production, also serves as a marker of oxidative stress (Lord & Bralley, 2008). Moreover, α-hydroxybutyric acid has been established as an early biomarker of insulin resistance and impaired glucose regulation (Gall et al., 2010; Sousa et al., 2021), and its elevated plasma levels have been reported in certain cancers (Tiziani et al., 2009; Zeng et al., 2014). Notably, in vivo studies show that α-hydroxybutyric acid not only promotes insulin resistance in a type 2 diabetes mouse model but also exacerbates tumor development in a colorectal cancer mouse model, suggesting that elevated α-hydroxybutyric acid may be a shared metabolic risk factor linking cancer and diabetes (Lv et al., 2023). Here, we observed higher α-hydroxybutyric acid levels in node-positive OSCC patients compared to node-negative patients. As lymph node metastasis is commonly associated with increased oxidative stress and enhanced antioxidant capacity (Tasdogan et al., 2021), elevated α-hydroxybutyric acid may reflect the metabolic reprogramming that facilitates tumor invasion and lymphatic dissemination. Nevertheless, given the exploratory nature of the analysis and the modest effect size with wide confidence intervals, our findings should be interpreted cautiously and do not establish a causal relationship between α-hydroxybutyric acid and metastatic potential. Given the close interplay between glucose dysregulation, oxidative stress and cancer progression (Giri et al., 2018), further studies in larger cohorts and functional models are required to clarify whether α-hydroxybutyric acid plays a mechanistic role in OSCC progression or represents a metabolic marker associated with disease severity.

A previous report showed that OSCC patients tend to have a low BMI, with the major tumor subsite being the floor of mouth, followed by the oral tongue (Polachini et al., 2023). This was, however, not seen in our patient cohort where the oral tongue was the predominant tumor subsite and SCCOT patients showed a high BMI with one-third being obese. Differences in the distribution of tumor subsites across studies may explain the discrepancies and suggest tumor subsite-specific metabolic alterations. Alternative metabolomic techniques with broader metabolite coverage will be required to investigate the potential for subsite-specific alterations.

Some limitations should, however, be acknowledged. First, differences in fasting status between patients and controls represent a potential source of pre-analytical variability. Second, the independent validation cohort lacked matched healthy controls, precluding formal external validation of classifier specificity and overall performance. Consequently, this cohort was used for descriptive validation only. Third, unmeasured factors such as undiagnosed metabolic conditions and recent dietary or drug intake may have influenced metabolite levels. Although exogenous drug-related features were excluded from downstream analyses, the potential impact of unrecorded medication or supplement use on endogenous metabolite levels cannot be completely excluded and therefore represents a common challenge in human metabolomics studies where complete control of medication and supplement use is often not feasible. Future studies with well-characterized cohorts are required to confirm the robustness and clinical utility of the identified markers. Finally, as metabolite annotations were assigned at MSI level-2, structural ambiguity may remain for certain metabolites, particularly for isomeric compounds that share identical mass-to-charge ratios and similar fragmentation patterns. Definitive confirmation would require comparison with authentic chemical standards or the use of additional orthogonal analytical approaches.

Nevertheless, our results reinforce the intricate connection between cancer, oxidative stress and glucose dysregulation and suggest a potential association between α-hydroxybutyric acid and metastasis. A deeper understanding of the relationships between glucose dysregulation and cancer could pave the way for improved prevention strategies, prognostic assessments, and novel therapeutic interventions in oral cancer management.

## Supplementary Information

Below is the link to the electronic supplementary material.

Supplementary Material 1

Supplementary Material 2

Supplementary Material 3

Supplementary Material 4

## Data Availability

The metabolomic data generated during this study is provided in a supplementary file.
