# Supplementary material for: Linking metabolism and metastasis: elevated α-hydroxybutyric acid in oral squamous cell carcinoma patients with lymph node metastasis
Source: Metabolomics. 2026 Apr 18;22(3):55. doi: 10.1007/s11306-026-02431-7 (PMC13091896; doi:10.1007/s11306-026-02431-7)
Supplement: Supplementary file 1 — Supplementary Material 1 [file 11306_2026_2431_MOESM1_ESM.docx]

Supplementary Information

**Linking Metabolism and Metastasis: Elevated α-Hydroxybutyric Acid in Node-Positive Patients with Oral Squamous Cell Carcinoma**

***Metabolomics***

Xiaolian Gu^1*^, Philip J Coates^2^, Lixiao Wang^1^, Nicola Sgaramella^1,3^, Mustafa Magan^1,4^, Karin Nylander^1^

^1^Department of Medical Biosciences/Pathology, Umeå University, 901 87, Umeå, Västerbotten, Sweden; ^2^Research Centre for Applied Molecular Oncology (RECAMO), Masaryk Memorial Cancer Institute, 656 53, Brno, Czech Republic; ^3^Department of Oral and Maxillo-Facial Surgery, Mater Dei Hospital, 701 25, Bari, Italy; ^4^Department of Clinical Sciences/ENT, Umeå University, Umeå, Västerbotten, 901 87, Sweden

***Correspondence to:** Xiaolian Gu, E-mail: [xiaolian.gu@umu.se](mailto:xiaolian.gu@umu.se)


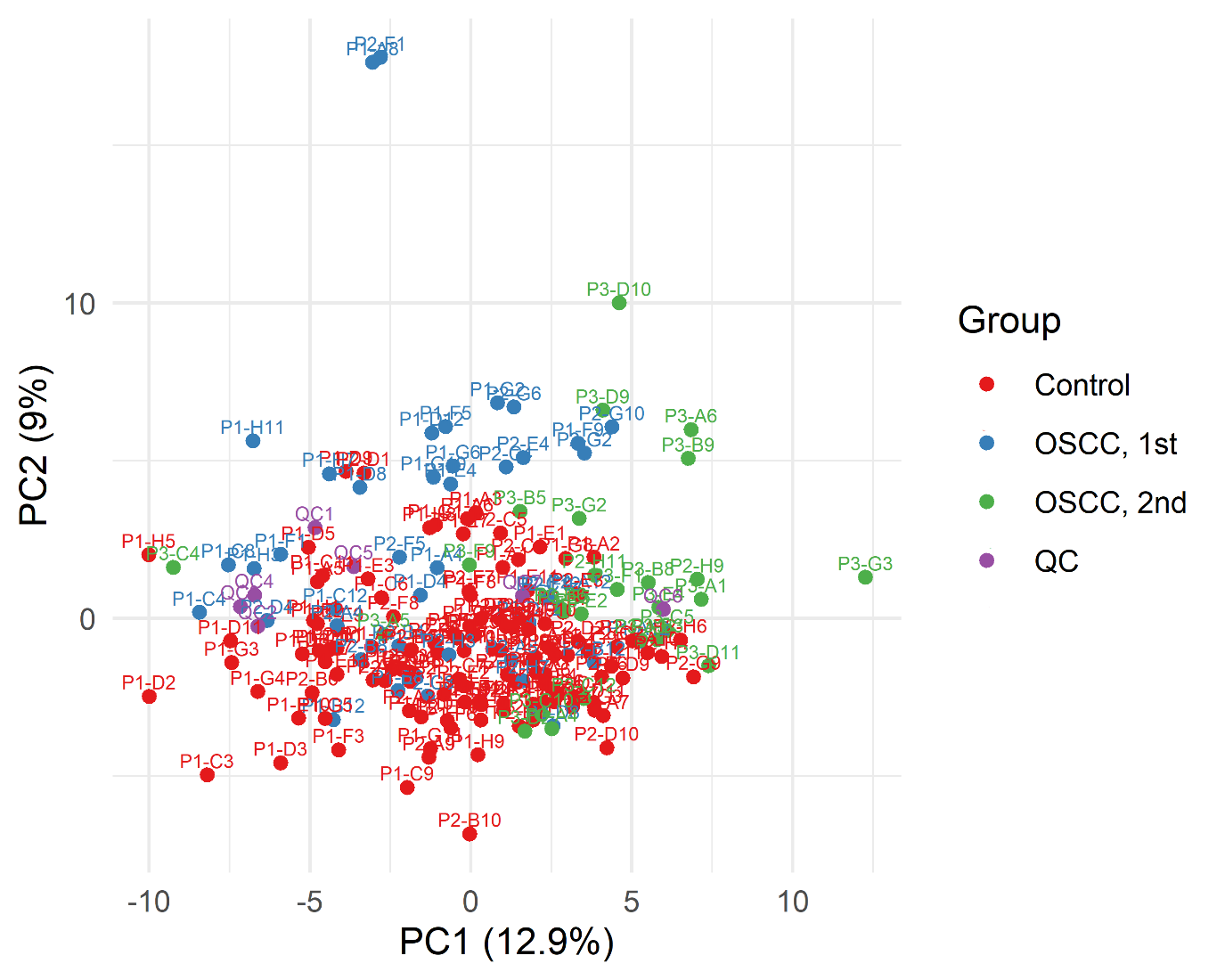


**Fig. S1**. Principal component analysis of metabolomic data, including seven quality control (QC) samples, showing sample distribution and QC clustering. OSCC, oral squamous cell carcinoma.

**
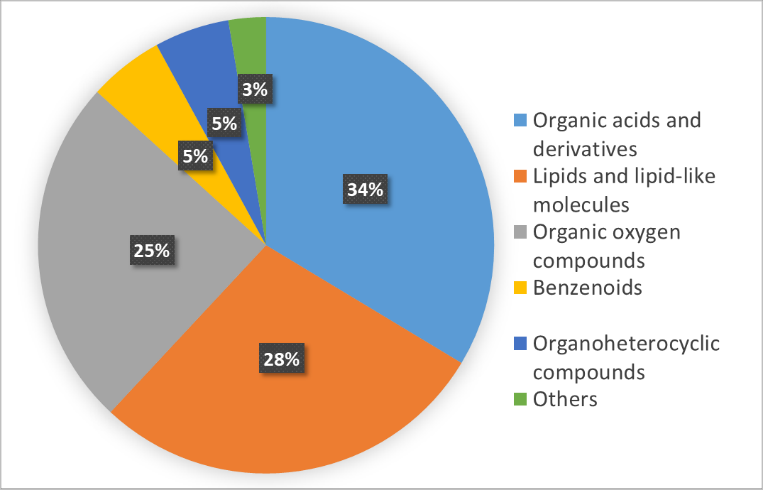
**

**Fig. S2** Classes and proportions of the 113 detected compounds


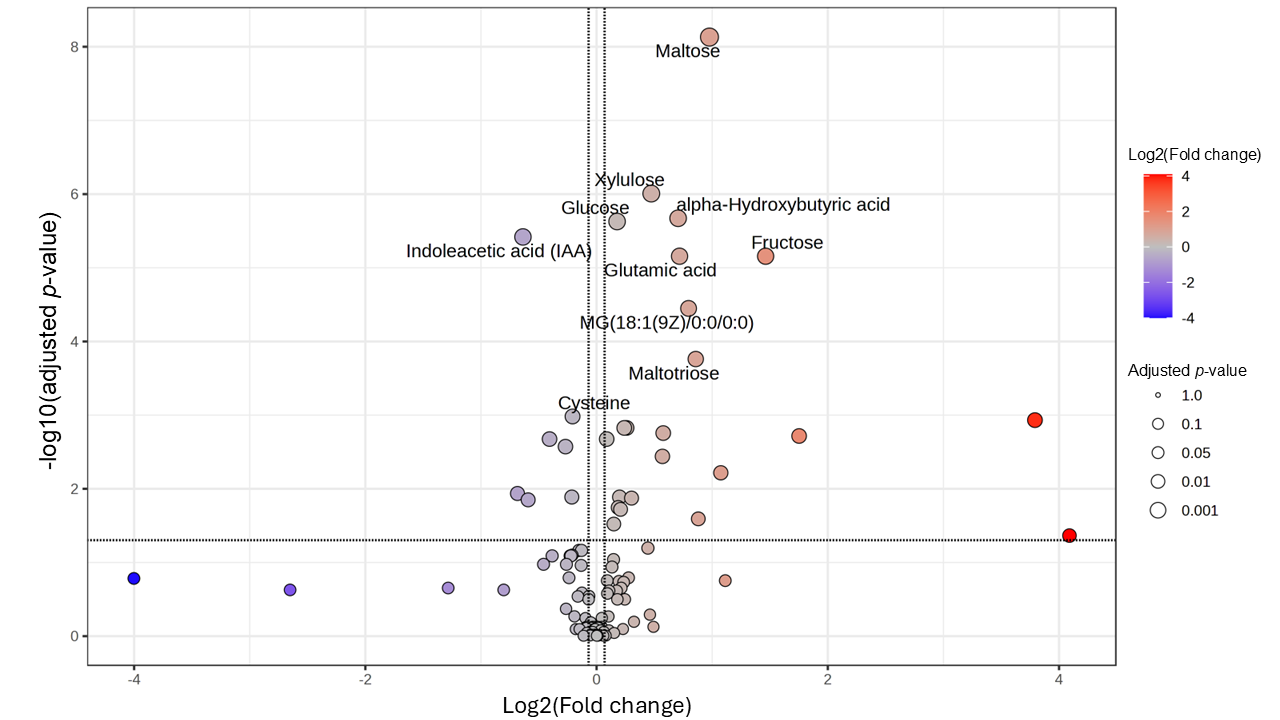


**Fig. S3** Volcano plot showing the overall differences in metabolite abundance between patients and matched controls. The names of the top 10 metabolites according to adjusted *p*-value were shown.


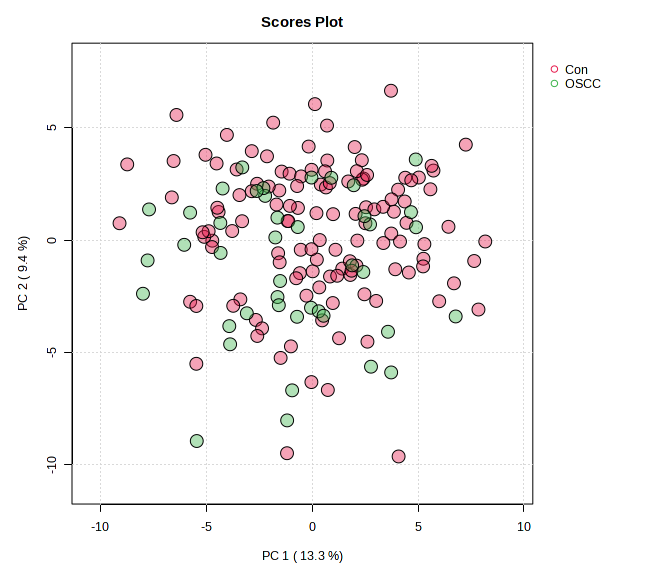


**Fig. S4** Principal component analysis of metabolomic data in patients and controls. Scores plot illustrating sample distribution based on the first two components derived from 103 metabolomic features. OSCC, oral squamous cell carcinoma. Con, matched cancer-free controls


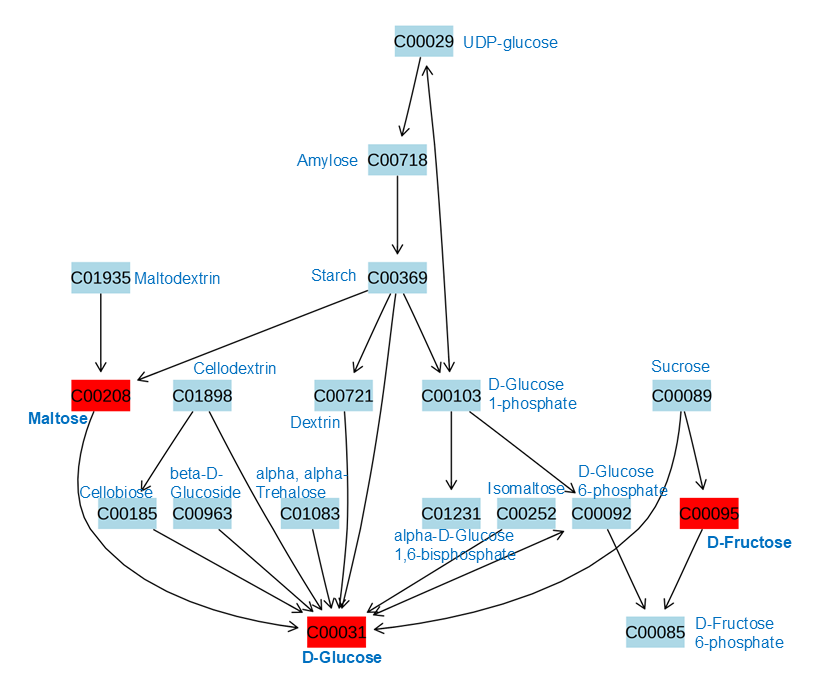


**Fig. S5** Starch and sucrose metabolism pathway. Figure generated using KEGG pathway analysis in MetaboAnalyst. Metabolites detected in this study are indicated, with their KEGG compound identifiers marked in red.
