# Supplementary material for: Linking metabolism and metastasis: elevated α-hydroxybutyric acid in oral squamous cell carcinoma patients with lymph node metastasis
Source: Metabolomics. 2026 Apr 18;22(3):55. doi: 10.1007/s11306-026-02431-7 (PMC13091896; doi:10.1007/s11306-026-02431-7)
Supplement: Supplementary file 3 — Supplementary Material 3 [file 11306_2026_2431_MOESM3_ESM.docx]

Supplementary Information

**Linking Metabolism and Metastasis: Elevated α-Hydroxybutyric Acid in Node-Positive Patients with Oral Squamous Cell Carcinoma**

***Metabolomics***

Xiaolian Gu1*, Philip J Coates2, Lixiao Wang1, Nicola Sgaramella1,3, Mustafa Magan1,4, Karin Nylander1

1Department of Medical Biosciences/Pathology, Umeå University, 901 87, Umeå, Västerbotten, Sweden; 2Research Centre for Applied Molecular Oncology (RECAMO), Masaryk Memorial Cancer Institute, 656 53, Brno, Czech Republic; 3Department of Oral and Maxillo-Facial Surgery, Mater Dei Hospital, 701 25, Bari, Italy; 4Department of Clinical Sciences/ENT, Umeå University, Umeå, Västerbotten, 901 87, Sweden

***Correspondence to:** Xiaolian Gu, E-mail: xiaolian.gu@umu.se

**Table S2** Comparison of classification performance across models using 5 to 100 features

|  | 5 features | 10 features | 15 features | 25 features | 50 features | 100 features |
| --- | --- | --- | --- | --- | --- | --- |
| Specificity | 0.864 | 0.872 | 0.88 | 0.872 | 0.904 | 0.888 |
| Sensitivity (Recall) | 0.857 | 0.881 | 0.833 | 0.833 | 0.881 | 0.81 |
| Accuracy | 0.862 | 0.874 | 0.868 | 0.862 | 0.898 | 0.868 |
| Precision | 0.679 | 0.698 | 0.7 | 0.686 | 0.755 | 0.708 |
| F1 score | 0.758 | 0.779 | 0.761 | 0.753 | 0.813 | 0.756 |
| MCC | 0.673 | 0.703 | 0.604 | 0.597 | 0.667 | 0.676 |

MCC: Matthews correlation coefficient

Definitions of performance metrics:

Specificity = TN / (TN + FP)

Sensitivity (recall) = TP / (TP + FN)

Accuracy = (TP + TN) / (TP + TN + FP + FN)

Precision = TP / (TP + FP)

F1 score = 2 × (Precision × Sensitivity) / (Precision + Sensitivity)

MCC = (TP × TN − FP × FN) / √[(TP + FP)(TP + FN)(TN + FP)(TN + FN)]

where TP, TN, FP, and FN denote true positives, true negatives, false positives, and false negatives, respectively.
