# Supplementary material for: Linking metabolism and metastasis: elevated α-hydroxybutyric acid in oral squamous cell carcinoma patients with lymph node metastasis
Source: Metabolomics. 2026 Apr 18;22(3):55. doi: 10.1007/s11306-026-02431-7 (PMC13091896; doi:10.1007/s11306-026-02431-7)
Supplement: Supplementary file 4 — Supplementary Material 4 [file 11306_2026_2431_MOESM4_ESM.docx]

Supplementary Information

**Linking Metabolism and Metastasis: Elevated α-Hydroxybutyric Acid in Node-Positive Patients with Oral Squamous Cell Carcinoma**

***Metabolomics***

Xiaolian Gu1*, Philip J Coates2, Lixiao Wang1, Nicola Sgaramella1,3, Mustafa Magan1,4, Karin Nylander1

1Department of Medical Biosciences/Pathology, Umeå University, 901 87, Umeå, Västerbotten, Sweden; 2Research Centre for Applied Molecular Oncology (RECAMO), Masaryk Memorial Cancer Institute, 656 53, Brno, Czech Republic; 3Department of Oral and Maxillo-Facial Surgery, Mater Dei Hospital, 701 25, Bari, Italy; 4Department of Clinical Sciences/ENT, Umeå University, Umeå, Västerbotten, 901 87, Sweden

***Correspondence to:** Xiaolian Gu, E-mail: xiaolian.gu@umu.se

**Table S3** Mann-Whitney U test *p*-values for metabolite–clinical feature comparisons

|  | | **Age** | **Sex** | **Fasting status** | **Tumor subsite** | **Tumor size** | **Nodal status** |
| --- | --- | --- | --- | --- | --- | --- | --- |
|  |  | **(Young vs.**  **Old)** | **(Female vs. Male)** | **(Fasting vs. Non-fasting)** | **(Oral tongue vs. Other oral subsites)** | **(T1,T2 vs. T3, T4)** | **(Negative vs. Positive)** |
| **Control** | **Maltose** | 0.203 | 0.316 |  |  |  |  |
|  | **Indoleacetic acid** | 0.136 | 0.528 |  |  |  |  |
|  | **Xylulose** | 0.002 | 0.118 |  |  |  |  |
|  | **Glucose** | 0.046 | 0.211 |  |  |  |  |
|  | **Glutamic acid** | 0.835 | 0.046 |  |  |  |  |
|  | **Fructose** | 0.782 | 0.030 |  |  |  |  |
|  | **Cysteine** | 0.010 | 0.230 |  |  |  |  |
|  | **α-hydroxybutyric acid** | 0.128 | 0.824 |  |  |  |  |
|  | **δ-gluconolactone** | 0.519 | 0.025 |  |  |  |  |
|  | **MG(18:1(9Z)/0:0/0:0)** | 0.201 | 0.984 |  |  |  |  |
| **1st patient cohort** | **Maltose** | 0.295 | 0.609 | 0.235 | 0.946 | 0.823 | 0.927 |
|  | **Indoleacetic acid** | 0.634 | 0.276 | 0.721 | 0.206 | 0.555 | 0.609 |
|  | **Xylulose** | 0.027 | 0.865 | 0.399 | 0.775 | 0.885 | 0.555 |
|  | **Glucose** | 0.037 | 0.927 | 0.898 | 0.321 | 0.684 | 0.703 |
|  | **Glutamic acid** | 0.903 | 0.101 | 0.007 | 0.924 | 0.906 | 0.203 |
|  | **Fructose** | 0.634 | 0.627 | 0.310 | 0.271 | 0.590 | 0.969 |
|  | **Cysteine** | 0.362 | 0.423 | 0.898 | 0.014 | 0.325 | 0.503 |
|  | **α-hydroxybutyric acid** | 0.673 | 0.014 | 0.368 | 0.105 | 0.487 | **0.061** |
|  | **δ-gluconolactone** | 1.474E-04 | 0.153 | 0.558 | 0.596 | 0.763 | 0.723 |
|  | **MG(18:1(9Z)/0:0/0:0)** | 0.100 | 0.684 | 0.637 | 0.989 | 0.969 | 0.503 |
| **2nd patient cohort** | **Maltose** |  | 0.698 |  | 0.923 | 1.000 | 0.643 |
|  | **Indoleacetic acid** |  | 0.808 |  | 0.089 | 0.225 | 0.217 |
|  | **Xylulose** |  | 0.225 |  | 0.528 | 0.007 | 0.007 |
|  | **Glucose** |  | 0.225 |  | 0.846 | 0.109 | 0.123 |
|  | **Glutamic acid** |  | 0.734 |  | 0.382 | 0.207 | 0.504 |
|  | **Fructose** |  | 0.593 |  | 0.662 | 0.264 | 0.040 |
|  | **Cysteine** |  | 0.497 |  | 0.332 | 0.037 | 0.918 |
|  | **α-hydroxybutyric acid** |  | 0.438 |  | 0.207 | 0.023 | **0.045** |
|  | **δ-gluconolactone** |  | 0.808 |  | 0.497 | 0.159 | 0.051 |
|  | **MG(18:1(9Z)/0:0/0:0)** |  | 0.698 |  | 0.244 | 0.099 | 0.382 |
